# A widespread alternate form of cap-dependent mRNA translation initiation

**DOI:** 10.1038/s41467-018-05539-0

**Published:** 2018-08-03

**Authors:** Columba de la Parra, Amanda Ernlund, Amandine Alard, Kelly Ruggles, Beatrix Ueberheide, Robert J. Schneider

**Affiliations:** 1 Department of Microbiology, NYU School of Medicine, New York, NY 10016 USA; 2 Perlmutter Cancer Center, NYU School of Medicine, New York, NY 10016 USA; 3 Department of Medicine, NYU School of Medicine, New York, NY 10016 USA; 4 Department of Biochemistry and Molecular Pharmacology, NYU School of Medicine, New York, NY 10016 USA

## Abstract

Translation initiation of most mammalian mRNAs is mediated by a 5′ cap structure that binds eukaryotic initiation factor 4E (eIF4E). However, inactivation of eIF4E does not impair translation of many capped mRNAs, suggesting an unknown alternate mechanism may exist for cap-dependent but eIF4E-independent translation. We show that DAP5, an eIF4GI homolog that lacks eIF4E binding, utilizes eIF3d to facilitate cap-dependent translation of approximately 20% of mRNAs. Genome-wide transcriptomic and translatomic analyses indicate that DAP5 is required for translation of many transcription factors and receptor capped mRNAs and their mRNA targets involved in cell survival, motility, DNA repair and translation initiation, among other mRNAs. Mass spectrometry and crosslinking studies demonstrate that eIF3d is a direct binding partner of DAP5. In vitro translation and ribosome complex studies demonstrate that DAP5 and eIF3d are both essential for eIF4E-independent capped-mRNA translation. These studies disclose a widespread and previously unknown mechanism for cap-dependent mRNA translation by DAP5-eIF3d complexes.

## Abstract (web summary)

Binding of eIF4E to the 5′ cap of mRNAs is a key early step in canonical translation initiation, but the requirement for eIF4E is not universal. Here the authors show that the eIF4G homolog DAP5 interacts with eIF3 to promote cap-dependent translation of a significant number of mRNA in an eIF4E-independent manner.

## Introduction

Translation of most mRNAs is controlled at the rate-limiting step of initiation, involving formation of a pre-initiation complex seeded by recognition of the m^7^GTP “cap” recognized by cap-binding protein eukaryotic initiation factor (eIF)4E, which recruits scaffolding protein eIF4G, ATP-dependent RNA helicase eIF4A, the multi-subunit complex eIF3, and the 40S ribosome subunit, among other proteins^1,2^. eIF4E-cap interaction is thought to be rate-limiting, as eIF4E is typically less abundant and the majority of mRNAs are cap-dependent. eIF4E abundance and mRNA translation are coordinately regulated by eIF4E sequestration through the eIF4E-binding proteins (4E-BPs)^1^. Nevertheless, quantitative sequestration of eIF4E by the 4E-BPs, or its strong reduction by small interfering RNA (siRNA) silencing, reduces but by no means extinguishes protein synthesis, and a great many mRNAs continue to translate^3–7^. While a small number of mRNAs support an alternate form of translation initiation that is independent of eIF4E and the cap, known as internal ribosome entry site (IRES)-mediated translation, this can only account for translation of several percent of mRNAs^4,8,9^.

eIF4G consists of three protein family members (Supplementary Fig. 1): eIF4GI (major form, highest expression, gene: *EIF4GI*); eIF4GII (minor form, lowest expression, gene: *EIF4G3*); and the poorly studied homolog, DAP5 (also known as NAT1, eIF4G2, and p97, gene: *EIF4G2*). DAP5 is homologous to the middle and C-terminal two-thirds of eIF4GI and can therefore bind eIF4A and eIF3 but cannot bind eIF4E and PABP because it lacks the N-terminal domain^10–13^. Because DAP5 lacks the N-terminal domain for eIF4E and PABP binding, studies have revealed that DAP5 can promote alternative translation of mRNAs that utilize eIF4E-cap-independent mechanisms by IRES elements. Some of these mRNAs are specifically translated during invasion, metastasis, cell progression, and apoptosis, and include Bcl-2, Apaf-1, cIAP1, CDK1, and p53^14,15^. Recent genome-wide translation profiling studies in embryonic stem cells have shown that DAP5 is involved in the translation of proteins required for cell differentiation^16,17^.

While DAP5 can promote IRES-dependent mRNA translation^12,14,15^, we and others have previously reported that with silencing of DAP5, there is an approximately 20% reduction in overall protein synthesis^3,12,18,19^, which far exceeds the several percent of cellular mRNAs thought to utilize internal ribosome initiation through IRES elements. We therefore asked whether DAP5 might promote a widespread alternate form of cap-dependent, but eIF4E-independent mRNA translation.

Here we used a comprehensive genome-wide transcriptome and translatome analysis of cells silenced in DAP5 and identified a significant fraction of mRNAs that are strongly reduced in translation with DAP5 deficiency. We show that highly DAP5-dependent mRNAs are enriched in those involved in cell death and survival, cell proliferation, cell mobility, DNA damage and repair responses, and translation initiation, and most do not contain IRESs. In all, approximately 20% of mRNAs were found to be strongly DAP5-dependent. Mass spectrometry analysis of DAP5, eIF4GI, and eIF4GII demonstrated that the recently identified novel cap-binding protein eIF3d is a direct binding partner of DAP5, whereas it is only weakly associated with eIF4GI and eIF4GII, likely through indirect association as a component of the eIF3 complex. In vitro translation and 48S complex isolation studies demonstrate that translation of these DAP5-dependent mRNAs is also co-dependent on eIF3d, which occurs through its direct interaction with DAP5. We propose that despite lacking eIF4E- and PABP-binding sites, DAP5 directs translation of many capped mRNAs, most of which do not possess an IRES element, but instead utilize a cap-dependent translation initiation process directed by DAP5-eIF3d.

## Results

### Translatomic analysis identifies DAP5-dependent mRNAs

**Fig. 1:**
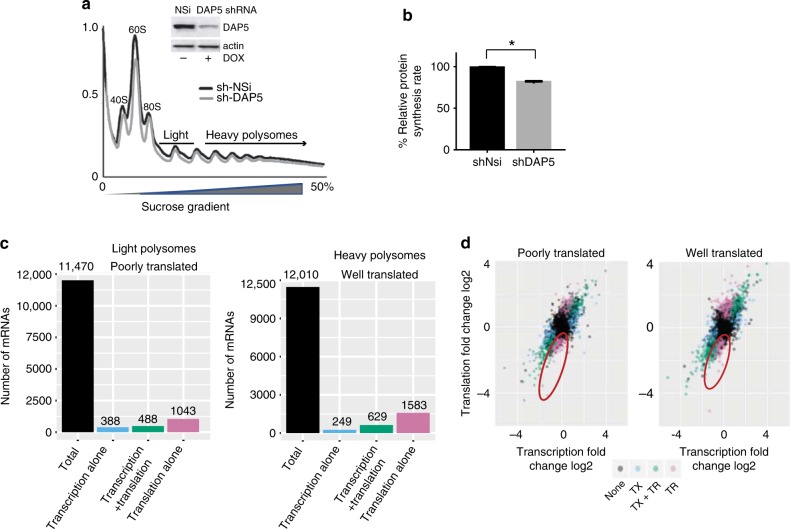
Genome-wide transcriptiomic and translatomic analysis of DAP5 dependence. **a** Ribosome absorbance profiles obtained from sucrose density gradient analysis of MDA-MB-231 cell lysates without and with DAP5 silencing by Dox-inducible TRIPZ RFP shRNA lentivirus vectors. Light (poorly translated) and heavy (well translated) polysomes and ribosome subunits (40S, 60S, and 80S) are indicated. DAP5 silencing MDA-MB-231 cells was confirmed by immunoblot (inset). **b** Relative protein synthesis rates determined by SUnSET assay-Puromycin incorporation. Puromycin incorporation signals were normalized to loading controls, quantified, and expressed as percent relative protein synthesis rates (*n* = 3). For source data and controls see Supplementary Fig. 2. *P < 0.05 by paired *t*-test. **c** Histogram representation of number of mRNAs out of total mRNAs altered in all three treatment conditions for transcription, transcription + translation, or translation alone (translation efficiency). **d** Total mRNA and purified mRNA from poorly and well-translated polysome fractions in (**a**) were subjected to RNAseq analysis. Log2 scatter plots shown for genome-wide transcriptomic and translatomic results of poorly translated and well-translated mRNAs, comparing non-silenced control to DAP5-silenced MB-231 cells. Data were analyzed for altered transcription alone (TX), combined transcription + translation (TX + TR), and translation alone (TR: translation efficiency = TR/TX for each mRNA). Two complete sets of independently performed studies were used to develop transcriptome and translatome data sets for analysis. For source data see Supplementary Data 1

**Fig. 2:**
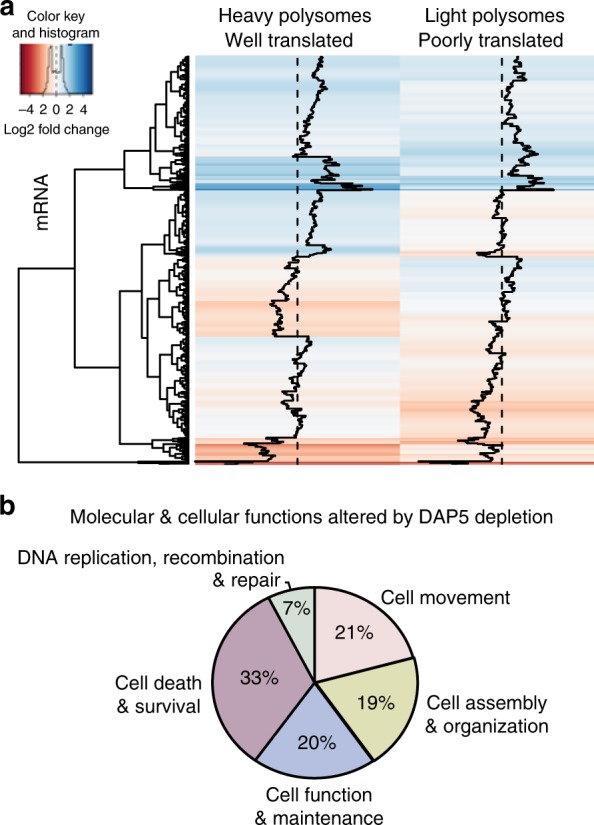
Transcriptomic and translatomic analysis of non-silenced and DAP5-silenced MDA-MB-231 cells. Total mRNA; light polysome, poorly translated mRNA (2–3 ribosome fraction); and heavy polysome, well-translated mRNA (≥4 ribosome fraction) were subjected to RNAseq. Average of two independent complete studies shown. **a** Heat map of RNAseq-mRNAs dependent on DAP5 for well-translated fractions. The black lines indicate the average fold change per gene across the dataset. Polysome mRNAs most strongly reduced with DAP5 silencing can be found in Supplementary Data 1 for source data. **b** Top predicted cellular functions affected with DAP5 silencing determined by Ingenuity Pathway Analysis (IPA)

Genome-wide transcription and translation analyses were performed to identify mRNAs whose translation is highly sensitive to reduction in DAP5 levels. MDA-MB-231 breast cancer cells were silenced with doxycycline (Dox)-inducible short hairpin RNAs (shRNAs) targeting DAP5 (MB-231-shDAP5) or a non-silencing control (MB-231-shNSi). Profiles showed a slight decrease in polysome content in DAP5-depleted cells (Fig. 1a), consistent with a 20–30% reduction in overall protein synthesis (Fig. 1b; Supplementary Fig. 2). RNA-sequencing (RNAseq) was carried out on total mRNA, poorly translated mRNA (2–3 light polysome fraction), and well-translated mRNA (≥4 heavy polysome fraction). mRNAs were identified that changed in abundance alone (largely transcription), translation alone (translation efficiency, ratio of mRNA in polysomes/total mRNA), or transcription + translation (Fig. 1c). By far the major changes with DAP5 silencing in light and heavy polysomes (poorly and well-translated mRNAs) were in the fraction altered solely in translation (translation efficiency). Approximately 9% of poorly translated mRNAs and 13% of well-translated mRNAs were found to be DAP5-dependent. Normalization of mRNA in polysomes to total mRNA was used to derive translation efficiency (Table 1). There were strong reductions in translation efficiency shown by distribution plots comparing *P*-values (*P* < 0.05) across log_2_ fold changes (Fig. 1d). The strong reductions in translation efficiency were particularly notable when viewed in heat map distribution plots (Fig. 2a).

**Table 1:** mRNAs most strongly reduced in polysomes with DAP5 silencing

Functions	Factor	mRNA predicted to have an IRES
Transcription factor involved in differentiation, proliferation, angiogenesis, apoptosis, tumor vascularization, and invasion	ETS1—proto-oncogene 1 transcription factor	NO
Transcription factor critical for normal development and oncogenesis	Fli-1—proto-oncogene, ETS transcription factor	NO
Cell migration/invasion and angiogenesis	OSMR—oncostatin M receptor	NO
Tissue development, cell invasion, and metastasis	LAMC1—laminin subunit gamma 1	NO
Angiogenesis and cell migration	SERPINE2—serpin family E member 2	NO
DNA damage and repair response	TP53 BP1—tumor protein p53-binding protein 1	NO
Cell proliferation, differentiation, survival	MYC-L—proto-oncogene, BHLH transcription factor (L-Myc protein)	NO
Signaling receptors	ITGV—integrin subunit alpha V	NO
	ITGα1—integrin subunit alpha 1	NO
	ITGα3—integrin subunit alpha 3	NO
	ITGα5—integrin subunit alpha 5	NO
Growth factor receptor that induces cell differentiation and proliferation	EGFR—epidermal growth factor receptor	NO
Regulates expression of genes involved in DNA repair	CDK12—cyclin-dependent kinase 12	NO
Breakdown of the extracellular matrix, tissue remodeling, cell migration	MMP1^a^ MMP3^a^ matrix—metalloproteinase 1 and 3	YES
Member of the transcription factor activator protein (AP)-1	JUN^a^	YES

IRES predictors: IRESite and IRESPred. Computational tool to predict the presence of internal ribosome entry site (IRES) in viral and cellular sequences

^a^Transcripts transcriptionally and translationally changing with DAP5 silencing

Ingenuity Pathway Analysis (IPA) was used to develop categorical classifications from the data shown in Figs. 1d and 2a. mRNAs most strongly reduced in translation with DAP5 silencing, independent of their steady-state mRNA abundance, were highly enriched in cell death and survival, cellular assembly and organization, cellular mobility, and DNA repair (Fig. 2b; Table 1). Of particular interest, DAP5-dependent mRNAs were enriched in key pro-oncogenic transcription factor and cell receptor mRNAs that are involved in cell proliferation, angiogenesis, anti-apoptosis, tumor invasion, and other wound-healing and oncogenic activities. Among mRNAs found to be highly DAP5-dependent for their translation were those encoding transcription factor ETS1 and some of its downstream mRNA targets, including those encoding laminin subunit gamma 1 (LAMC1), matrix metalloproteinases (MMP1 and 3), serpin family E member 2 (SERPINE2), and a number of pro-oncogenic integrin subunit proteins. Similar autologous transcription-translation expression loops were identified for the pro-oncogenic MYC-L transcription factor mRNA, and mRNAs for cell receptors such as oncostatin M (OSMR), and the epidermal growth factor receptor (EGFR), among others (Table 1 and Supplementary Data 1). Of the mRNAs dependent on DAP5 for translation, few have been shown to utilize, or are predicted to utilize IRES-mediated translation initiation.

### DAP5 binds directly and strongly with eIF3d

**Fig. 3:**
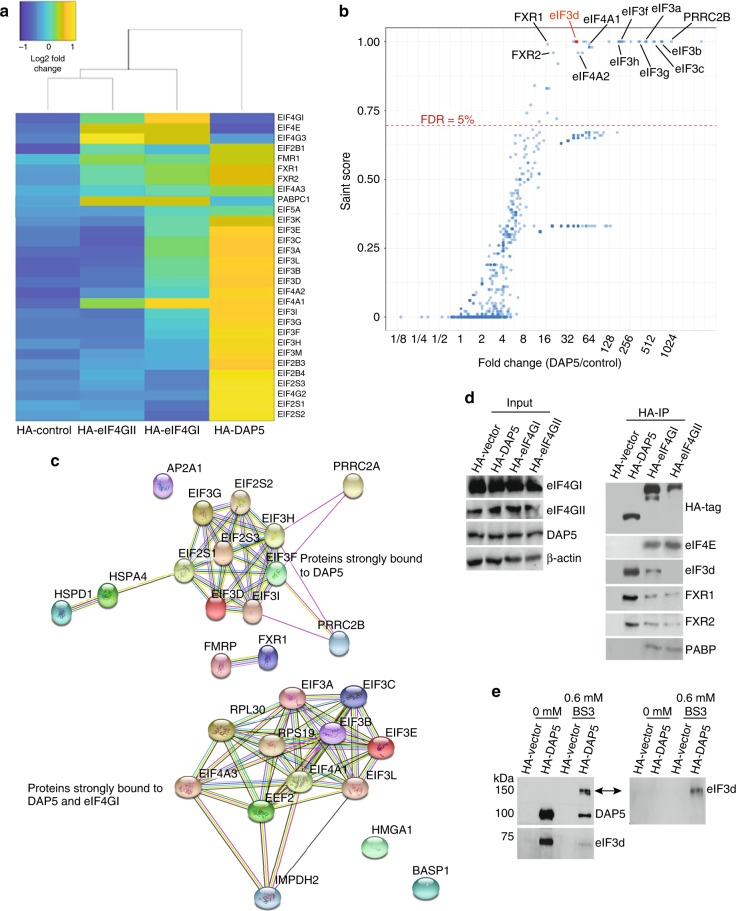
Proteomic analysis of the eIF4G family. **a** Heat map of top-ranked interacting proteins with DAP5, and/or eIF4GI, eIF4GII, and HA-control (HA protein alone). Cluster analysis carried out based on the intensity and coverage of proteins by mass spectrometry (LC-MS). Yellow and blue, respectively, represent strongest and weakest binding proteins to eIF4GI, eIF4GII, and/or DAP5 (*n* = 3). **b** eIF3d identified as one of the strongest DAP5-binding partners. SAINT score was plotted against the ratio of spectral counts in the DAP5 immunoprecipitation (IP) over control (HA-IP). Five percent FDR cutoff shown. Protein identification results are labeled for the MS analysis of affinity purification (*n* = 3) for DAP5 using the SAINT algorithm. **c** Data analysis using SAINT algorithm of the top-ranked protein interactions (SAINT score > 0.7): (top) proteins interacting exclusively with DAP5; and (bottom) proteins interacting in common with DAP5 and eIF4GI. See Supplementary Data Fig. 3 for STRING analysis of eIF4GI, eIF4GII-interacting proteins, and Supplementary Data 2 for source data. **d** IP and immunoblot analysis validation of HA-DAP5-, HA-eIF4GI-, and HA-eIF4GII-interacting proteins. **e** In vivo crosslinking with bis(sulfosuccinimidyl) BS3 before cell lysis and IP of HA-DAP5. Immunoblot analysis confirms crosslinking of DAP5 and eIF3d by appearance of band in SDS-denaturing gel at higher molecular weight > 150 kDa (arrows), identified by anti-DAP5 and anti-eIF3d antibodies only from cells treated with BS3. Membrane was first immunoblotted with anti-DAP5, then stripped and re-probed for anti-eIF3d

Biochemical studies have shown that DAP5 interacts with eIF4A, eIF3, the MNK (eIF4E) kinases, and eIF2β^10,11,16^. We therefore sought to identify novel interacting proteins of DAP5 using stringent immunoprecipitation (IP) followed by liquid chromatography-mass spectrometry (LC-MS) analysis, comparing the interacting partners and strength of interaction with DAP5, eIF4GI, and eIF4GII. Proteins were N-terminally tagged using hemagglutinin (HA) and expressed to the nearest-normal endogenous levels in MDA-MB-231 cells, along with empty vector controls. Protein quantitation was performed using the ratios of peptide spectral matches (PSMs) affinity purification (AP) over the PSMs in the control. In addition, the data were analyzed using the SAINT algorithm^20^ (Supplementary Data 2). DAP5, eIF4GI, and eIF4GII all interacted with eIF4A1, whereas DAP5 did not interact with eIF4E or PABP, as expected (Fig. 3a, b). The proteins characterized as specific interactors were subjected to analysis by STRING with eIF4GI and DAP5, or exclusively with DAP5 (Fig. 3c; Supplementary Data 2). STRING analysis of eIF4GI and eIF4GII are also shown (Supplementary Fig. 3).

In addition to expected interaction with known binding partners, including some eIF3 proteins, eIF4GI, DAP5, and eIF4GII interacted to varying extents with fragile-X FXR1 and FXR2 proteins, whose potential translation functions are only poorly understood (Fig. 3a–c; Supplementary Fig. 3). FXR1 was previously shown to complex with AGO2, miRs, and DAP5 in quiescent cells^21^. Collectively, these data demonstrate that only a relatively small number of translationally relevant interacting proteins are found in common among all three eIF4G family members. A total of 33 proteins involved in protein synthesis were found to interact in common between eIF4GI and DAP5, whereas eIF4GI and DAP5 each uniquely interacted with more proteins than they had in common (Supplementary Fig. 4).

Several proteins strongly and exclusively interacted with DAP5, or only very weakly with eIF4GI or eIF4GII (Fig. 3a, b, d; Supplementary Data 2). One of the strongest DAP5-interacting proteins was eIF3d. eIF3d was of particular interest because it was shown to have cap-binding activity but has only been shown to date for translation of transcription factor *c-Jun* mRNA^22,23^. IP analysis confirmed the lack of interaction between DAP5 and eIF4E/PABP, but strong interaction with eIF3d (and FXR1/2, FMR1) compared to weak binding of eIF3d to eIF4GI, and only very weak binding to eIF4GII. These data suggest that eIF3d interaction with eIF4GI and II is likely indirect via other eIF3 proteins, but direct with DAP5 (Fig. 3d). A direct interaction between DAP5 and eIF3d was confirmed using live cell high-specificity bifunctional chemical crosslinking with bis(sulfosuccinimidyl) suberate (BS3), followed by IP and immunoblot analysis (Fig. 3e). eIF3d was retained in an electrophoretically stable and larger complex with DAP5 despite denaturation conditions. eIF3d is therefore a direct protein-binding partner of DAP5.

### DAP5-eIF3d drives cap-dependent mRNA translation

**Fig. 4:**
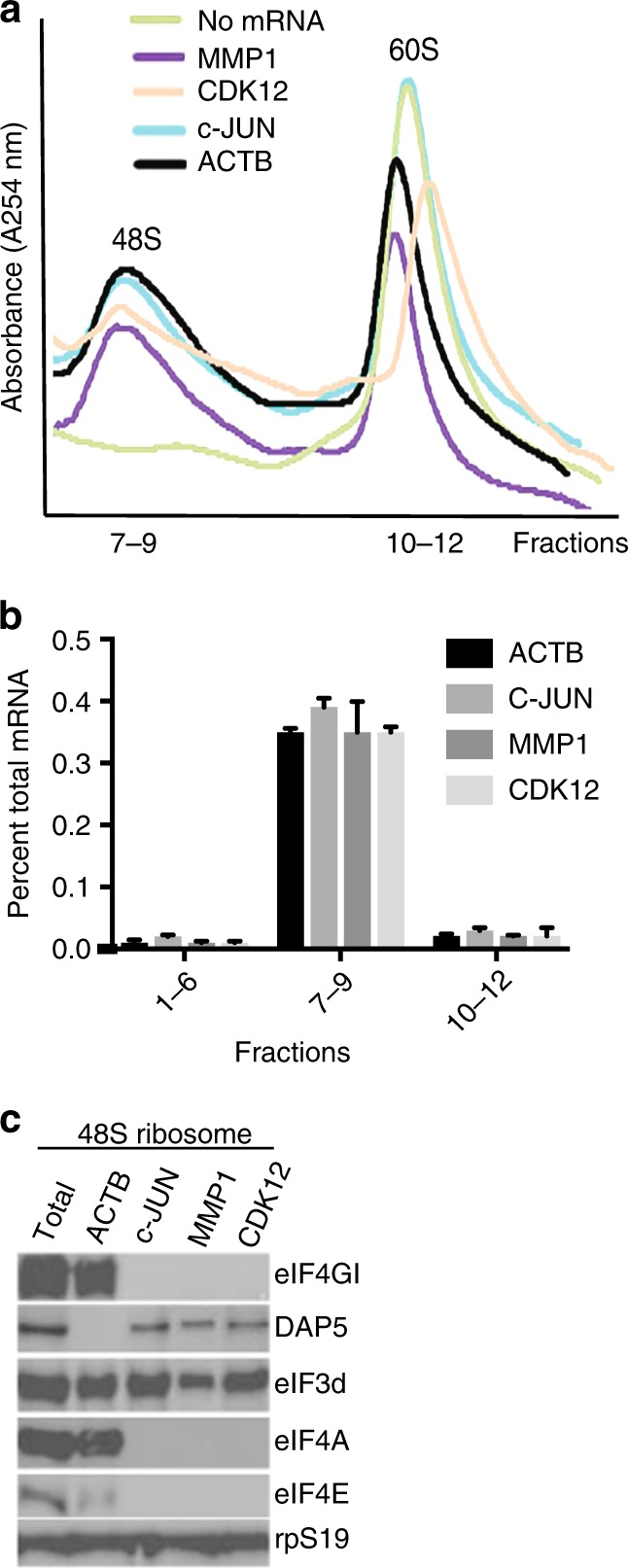
DAP5 interacts with eIF3d to drive selective translation independently of eIF4E and eIF4GI. **a** Distribution of mRNAs encoding c-JUN, ACTB, MMP1, and CDK12 in 48S ribosome-mRNA complexes in 293T cell in vitro mRNA programmed translation extracts (see Supplementary Fig. 5 flow chart for methodology). In vitro translation extracts lacking endogenous mRNAs were programmed with in vitro-synthesized capped and polyadenylated mRNAs. 48S and 60S ribosome subunit distribution profiles from in vitro translation extracts were plotted by relative absorbance at 254 nm against elution fractions. Non-programmed extracts served as controls. **b** Quantitative RT–PCR analysis of the in vitro translation fractions shown in (**a**) expressed as relative mRNA abundance as a fraction of total recovered mRNA. **c** Immunoblot analysis of initiation factors in isolated 48S ribosome-mRNA translation complexes fractionated by sucrose gradients formed with mRNAs encoding c-JUN, ACTB, MMP1, or CDK12. Total protein is a control from non-programmed 293T cell in vitro translation extracts. Ribosomal protein S19 (rpS19) is a loading control (*n* = 3)

We therefore asked whether DAP5 is converted from a specialized translation factor for a small number of IRES-containing mRNAs into a widely used alternate cap-dependent translation initiator through direct binding to eIF3d. We performed in vitro translation using human 293T cell extracts programmed with capped and polyadenylated mRNAs encoding c-JUN, ACTB, MMP1, and cyclin-dependent kinase 12 (CDK12), which were found to require DAP5 in our genome-wide translatomic studies (Fig. 4a, b; Table 1). 48S mRNA-ribosome complexes were isolated by sucrose density gradient centrifugation. As reported^23^, none of the eIF4F factors (eIF4E, eIF4GI, and eIF4A1) were found in 48S complexes isolated with the *c-Jun* mRNA (Fig. 4c). However, DAP5 was detected in the 48S complex of DAP5-dependent mRNAs, including *c-Jun*, suggesting that the interaction of eIF3d-DAP5 is important for translation of these mRNAs. For ACTB, a canonical eIF4E-dependent mRNA, the eIF4F complex was detected, but without DAP5. These results indicate that DAP5 binds directly to eIF3d to facilitate selective translation independent of the eIF4F complex.

**Fig. 5:**
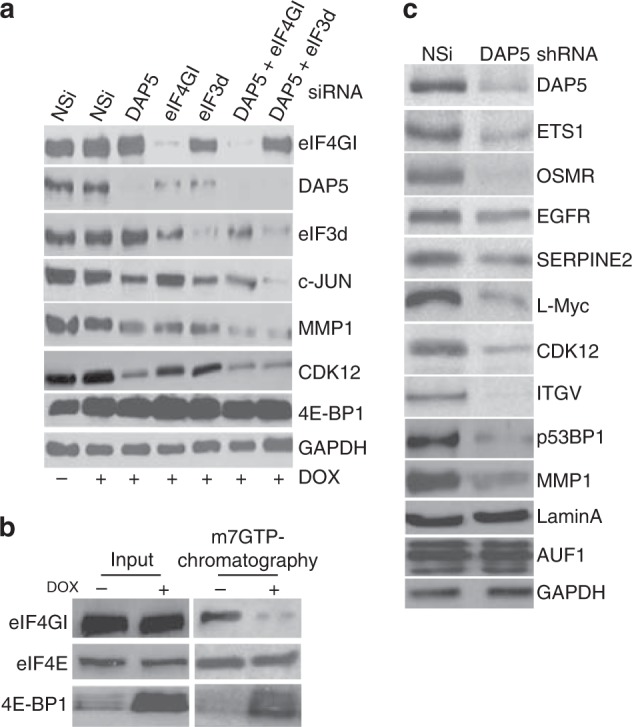
DAP5 and eIF3d are both required for translation of DAP5-dependent mRNAs. **a** Overexpression of 4E-BP1 by stable cDNA transformation in MDA-MB-231 cells silenced with siRNAs: non-silencing (NSi), DAP5, eIF4GI, eIF3d, DAP5 + eIF4GI, and DAP5 + eIF3d. **b** Cap chromatography of cell lysates with or without 4E-BP1 cDNA overexpression. Representative immunoblots show almost total sequestration of eIF4E by overexpressed 4E-BP1. **c** Representative immunoblots of MDA-MB-231 cells silenced with NSi or DAP5 siRNAs as in (**a**) probed for proteins whose mRNAs were found to be reduced in polyribosomes by DAP5 silencing (Table 1). Lamin A, AUF1, and GAPDH served as DAP5-independent controls. Results are representative of three independent experiments

To determine whether DAP5 and eIF3d are both necessary for the translation of DAP5- and eIF3d-dependent mRNAs, we silenced DAP5, eIF3d, or eIF4GI, individually and in combination (Fig. 5a). Cells were also engineered to inducibly overexpress 4E-BP1 to sequester eIF4E. In some cases cells were first silenced for 24 h with siRNAs then Dox-induced for 4E-BP1 overexpression. Overexpression of 4E-BP1 was found to abolish eIF4E-eIF4GI interaction and therefore eIF4F complex formation (Fig. 5b). Silencing eIF3d alone or DAP5 alone reduced levels of c-JUN by three- to fourfold, whereas co-silencing both DAP5 and eIF3d almost abolished protein levels of *c-Jun* (Fig. 5a). Silencing eF4GI had no effect on *c-Jun* protein levels. The protein levels of MMP1 and CDK12 were reduced by half with silencing eIF3d or DAP5, but almost abolished by co-silencing DAP5 and eIF3d. While silencing eIF4GI slightly reduced MMP1 and CDK12 levels, it should be noted that silencing eIF4GI also reduces DAP5 and eIF3d levels, which likely accounts for effects of eIF4GI silencing on DAP5 target mRNAs.

We also tested the effect of silencing DAP5 on the translation of other DAP5-dependent mRNAs identified by translatomic analysis. Immunoblot studies confirmed that protein levels of DAP5-dependent mRNAs are partially or completely inhibited by DAP5 silencing (Fig. 5c; Table 1; Supplementary Data 1). Of the DAP5-dependent mRNAs identified in our genome-wide studies, ETS1, OSMR, L-Myc, ITGV, p53-BP1, and MMP1 were strongly reduced with DAP5 silencing, whereas EFGFR and SERPINE2 were moderately downregulated. We also determined levels of proteins whose mRNAs were found not to be DAP5-dependent, including Lamin A, AUF1, and GAPDH (Fig. 5d). None of these protein levels were altered with DAP5 silencing, even for short-lived mRNAs such as AUF1.

## Discussion

Our results indicate that a surprising number of capped mRNAs require DAP5 for their translation, and do not contain IRES elements. DAP5 mRNA targets are particularly enriched in those encoding proteins implicated in cell survival, motility, and DNA repair. All of the DAP5-dependent mRNAs use, to varying extents, the interaction between DAP5 and eIF3d for their translation initiation. The mechanistic details by which DAP5-eIF3d and eIF4E-eIF4GI mRNA selectivity are determined are not yet understood. It is likely that certain mRNAs can use either eIF4E-eIF4GI or DAP5-eIF3d, possibly dependent on physiological context such as stress that inhibits mTOR and sequesters eIF4E. It is also likely the certain mRNAs can use both eIF4GI/eIF4E complexes and DAP5/eIF3d complexes equally well under the same physiological conditions. In this regard, ribosome profiling rather than polysome profiling might increase the list of mRNAs under DAP5-eIF3d control and inform the impact on upstream open reading frame (ORF) usage between DAP5/eIF3d-mediated and eIF4GI/eIF4E-mediated translation initiation. We also suspect that the use of different pathways of translation initiation, including the canonical eIF4E-eIF4GI cap-dependent translation, as well as IRES-dependent translation, are likely dependent on cell type and tissue specificity, which deserves further investigation.

Translation initiation is a well-established and critical regulatory point in gene expression. It has also emerged more recently as a remarkably plastic response to a variety of physiological changes by altering the type of translation initiation mechanism used, thereby dynamically reprogramming the types of mRNAs that are translated in response, for example, to stress, drug resistance, and transformation^3,24,25^. Understanding these different mechanisms is paramount, as they fundamentally underlie the manifestation of diseases as widely represented as cancer and autoimmunity. Our identification of the DAP5-eIF3d complex as an additional and widely used mechanism for cap-dependent translation of mRNAs, accounts for a dark matter area of protein synthesis: the mechanism by which many mRNAs translate during physiological conditions of mTOR inhibition and eIF4E depletion.

## Methods

### Uncropped immunoblot image data

See Supplementary Fig. 6 for uncropped images of key immunoblot data presented in this study.

### Cell culture and transfection

Metastatic variant of MDA-MB-231 (ER−) and 293T were obtained from American Type Culture Collection, authenticated by short tandem repeat profiling and routinely checked for mycoplasma contamination. Cells were propagated in Dulbecco’s modified Eagle medium (Invitrogen, Houston, TX) supplemented with 10% fetal bovine serum (Invitrogen) at 37 °C in 5% CO_2_. Cells were transfected with plasmids using Lipofectamine 2000 (Invitrogen) as described by the manufacturer.

### Plasmids

pcDNA4 T7 c-JUN and pcDNA4 T7ACTB plasmids used for in vitro transcription were provided by Dr. Amy Lee (Brandeis University, Waltham, MA)^22,23^. The c-JUN template was constructed by amplifying the ORF and 3′ untranslated region from human cDNA and joined together downstream of a T7 promoter by Gibson assembly into pcDNA4. After added the T7 promoter to ACTB by PCR amplification the fragment was inserted into pcDNA4.

CDK12 and MMP1 templates were generated by PCR amplification and addition of a T7 RNA polymerase promoter with high-fidelity DNA polymerase (NEB#M0491). The design primers containing the T7 promoter followed by sequences of interest are:

5′-CATATGTAATACGACTCACTATAGGATGCCCAATTCAGAGAGA-3′ and

5′-CGCGGCCGCAGTAAGGAACTCCTCTC-3′ for CDK12, and

5′-CATATGTAATACGACTCACTATAGGATGCACAGCTTTCCTCC-3′ and

5′-GGCGGCCGCAATTTTTCCTGCAGTTGAACC-3′ for MMP1.

Prior to its use as a template for in vitro transcription, the PCR products were analyzed by agarose-gel electrophoresis and purify by PCR Cleanup Kit (NEB#T1030).

### pTripz construct expression and siRNAs

shRNA cassettes were cloned into 5′-*Xho*I and 3′-*Eco*RI sites of tetracycline-inducible lentiviral pTRIPZ vector driving the expression of a TurboRFP fluorescent reporter (GE Dharmacon technology). The shRNA cassette sequences were as follows:

eIF4G2 (5′-TACCTCTAGTAATGGGCTTTA-3′), and non-silencing sequence control, Nsi (5′-AATTCTCCGAACGTGTCACGT-3′).

Stable cells lines were generated using puromycin selection, Dox 1–2 µg/ml, and sorted by red fluorescent protein expression.

A siRNA targeting human *EIF4GI*, *EIF4G2*, and *EIF3D* genes and an unrelated siRNA as a control (control siRNA) were purchased from Ambion. Target sequences were:

5′-GGCAUACUAAAUAAGCUUATT-3′(siEIF4G2),

5′-CAUUCGUCGCUGAAACAGAATT-3′ (EIF4GI), and

5′-GAACCUCCGCAGAGACAAATT-3′ (EIF3D)

MDA-MB-231 Dox-inducible 4E-BP1 cells were transiently transfected with the lowest effective concentration of siRNA derived from titration analysis, which was 25 nM of siRNA target gene or siRNA control using TransIT-SiQUEST (Mirus Bio LLC) according to the manufacturer’s instructions. After 24 h of siRNA transfection, Dox (1 µg/ml) was added to the cells for 48 h to overexpress 4E-BP1. The pTRIPZ plasmid was modified to insert the cDNA for 4E-BP1 under the Dox-inducible promoter using *Age*I and *Mlu*I restriction sites.

### Puromycin incorporation (SUnSET) assay

Puromycin (10 μg/ml) was added to MDA-MB-231 cells expressing Dox-inducible shRNAs targeting DAP5 (MDA-MB-231-shDAP5) or control (MDA-MB-231-shNSi) and incubated for 10 min. Cells treated with cycloheximide (CHX; 100 μg/ml) were positive controls; cells that were not exposed to puromycin were negative controls. Cells were lysed and subjected to immunoblot analysis. Puromycin incorporation in neo-synthesized proteins (a measure of the rate of mRNA translation in vitro) was assessed with an anti-puromycin antibody (12D10 monoclonal antibody). The integrated density of positive bands was quantified using ImageJ software.

### Immunoprecipitation

MDA-MB-231 cells were transiently transfected with pcDNA3-HA-DAP5, pcDNA3-HA-eIF4GI, pcDNA3-HA-eIF4GII, or empty vector control. Cells were lysed in mild buffer (50 mM TrisHCl (pH 7.5), 150 mM NaCl, 0.5% NP-40, and complete protease inhibitor cocktail without dithiothreitol (DTT). Benzonase (25 U/ml) and RNase A (100 µg/ml) (EMD, Gibbstown, NJ) were added in the buffer. Proteins were eluted from beads with HA-peptide (Sigma). Eluates were submitted to the Proteomic Facility at NYU.

### Mass spectrometry sample preparation and data analysis

Samples from immunoprecipitates were reduced, alkylated, and loaded onto SDS-polyacrylamide gel electrophoresis gels to remove LC-MS incompatible reagents. Gel plugs were excised, destained, and subjected to proteolytic digestion with trypsin and resulting peptides extracted and desalted, as previously described^26^. Aliquots of the peptides were analyzed with LC-MS using a 60 min gradient on an EASY nLC 1000 coupled to a ThermoFisher Scientific Orbitrap Elite Hybrid Ion Trap Mass Spectrometer. The data were searched against a UniProt human database using Sequest within Proteome Discoverer. The results were filtered to only include proteins identified by at least two peptides. Protein quantitation was preformed using the ratios of PSMs in DAP5, eIF4GI, and eIF4GII AP over the PSMs in the control AP. In addition, the data were analyzed using the SAINT algorithm^27^, including experiments 52–54 from the crapome.org database^28^ as additional controls.

### Polysome-associated mRNA isolation and RNAseq

Polysome isolation was performed by separation of ribosome-bound mRNAs via sucrose gradient. Briefly, Beckman Ultra-Clear centrifuge tubes were loaded with 5.5 ml of 50% and 15% sucrose respectively in low-salt buffer [200 mM Tris (pH 7.4) in DEPC H2O, 100 mM NaCl, and 30 mM MgCl2] with 1:1000 RNasin (Fermentas) and 100 μg/ml CHX in ethanol and incubated at 4 °C horizontally overnight. MDA-MB-231 cells expressing Dox-inducible shRNAs targeting DAP5 (MDA-MB-231-shDAP5) or control (MDA-MB-231-shNSi) were pre-treated with 100 μg/ml CHX (Calbiochem), washed twice in phosphate-buffered saline (PBS) with 100 μg/ml CHX, pelleted, and resuspended in 700 μl of polysome isolation buffer (200 mM Tris (pH 7.5), 100 mM NaCl, and 30 mM MgCl_2_) with 1:1000 RNasin (Fermentas) and 100 μg/ml CHX in ethanol. After 5 min of incubation, 250 μl of detergent buffer (1.2% Triton, and 0.2 M sucrose in polysome isolation buffer) was added^18,29^, cells lysed, clarified lysates then layered onto 10–50% sucrose gradients (Sigma-Aldrich) and sedimented at 36 000 rpm for 2 h in a SW40 rotor (Beckman Coulter) at 4 °C. Gradients were collected in 15 × 750 μl fractions by pumping 60% sucrose into the bottom of the gradient and collecting from the top using an ISCO fraction collector while simultaneously monitoring absorbance at 254 nm. RNA was isolated by extraction using RNeasy Mini Kit (Qiagen) as per the manufacturer’s instructions. Fractions 4–12, representing well-translated polysomes were pooled and classified as light (poorly translated) polysome fractions (2–3 ribosomes) and well-translated heavy polysome fractions (≥4 ribosomes). RNA quality and amount were determined by the Agilent Technologies kit and Nanodrop. RNAseq was carried out by the NYU School of Medicine Genome Technology Core using the Illumina Hi-Seq 2500 Single Read. To quantify translational efficiency, the difference in log_2_ intensity between matched polysomal mRNA and total mRNA was determined. To examine differences in transcription and translation, total mRNA and polysome mRNAs quantile normalized independently. Statistical analysis was performed using the limma R package^30^. Gene enrichment analysis was performed using IPA software (QIAGEN Inc., https://www.qiagenbioinformatics.com/products/ingenuitypathway-analysis).

### BS3 crosslinking

Cells were harvested, washed with PBS, and incubated with 0.6 mM of BS3 (bis[sulfosuccinimidyl] suberate) for 30 min at room temperature. The reaction was quenched by adding 1 M Tris to a final concentration of 20 mM and incubated for 15 min at room temperature before the samples were lysed and immunoprecipitated (see above).

### Immunoblot antibodies

Immunoblot studies were performed using the following antibodies at 1:1000 dilution: anti-DAP5 (BD Biosciences 610742); anti-eIF3d (Bethyl A301-758A); anti-eIF4A1 (Cell Signaling 2490); anti-eIF4G1 (Cell Signaling 2858); anti-rpS19 (Bethyl A304-002A); anti-eIF4E (Bethyl A301-154A); anti-HA epitope tag (Abcam 18181); anti-c-Jun (Cell Signaling 9165); anti-CDK12 (Cell Signaling 11973); anti-MMP1 (Abcam 137332); anti-GAPDH (Cell Signaling 2118S); anti-4E-BP1 (Cell Signaling 9644); anti-Lumin A (Abcam 226189); anti-AUF1 (EMD Millipore 07260); anti FXR1 (Cell Signaling 12295); anti FXR2 (Cell Signaling 7098); and anti-PABP (Cell Signaling 4992). The following antibodies were used at 1:500 dilution: anti-ETS1 (Abcam 26096); anti-OSMR (Abcam 210771); anti-EGFR (Cell Signaling 4257); anti-SERPINE2 (Abcam 75348); anti-ITGV (Abcam 124968); and anti-p53-BP1 (Abcam 21083).

### In vitro transcription

RNAs were generated by in vitro transcription with T7 RNA polymerase (NEB), performed in the presence of 7-methylguanosine cap structure (NEB M0276), using linearized plasmid or PCR products as the template, and polyadenylated using polyA polymerase (NEB M2080S). RNAs were purified by phenol–chloroform extraction and ethanol precipitation.

### In vitro translation

In vitro translation extracts were made from human 293T cells as described^23^. Lysates were nuclease-treated with 18 gel U/μl micrococcal nuclease (NEB M0247S) in the presence of 0.7 mM CaCl_2_ for 10 min at 25 °C, and the digestion was stopped by addition of 2.24 mM EGTA. Each translation reaction contained 50% in vitro translation lysate (from 293T cells) and buffer to make the final reaction 0.84 mM ATP, 0.21 mM GTP, 21 mM creatine phosphate (Roche), 45 U/ml creatine phosphokinase (Roche), 10 mM HEPES-KOH, pH 7.6, 2 mM DTT, 8 mM amino acids (Promega), 255 mM spermidine, 1 U/ml murine RNase inhibitor (NEB), and mRNA-specific concentrations of Mg(OAc)2 and KOAc. For 48S ribosome subunit initiation complex purification from in vitro translation reactions, reactions were incubated in the presence of GMP-PNP for 20 min at 30 °C and centrifuged for 6 min at 12 000 × *g* at 4 °C. Lysates were purified by size-exclusion chromatography through a 1 ml column packed with Sephacryl S-400 gel filtration resin (GE Healthcare) and the eluant centrifuged through a 10–25% (w/v) sucrose gradient by centrifugation for 5 h at 36 000 rpm at 4 °C in a Beckman SW40 Ti rotor. Fractions were collected from the gradient and RNA purified by phenol–chloroform extraction and ethanol precipitation, and protein precipitated with trichloroacetic acid.

### Statistical analysis

Statistical analyses used the two-tailed Student’s *t*-test unless otherwise noted, with *P* < 0.05 taken as significance.

### Data availability

Data supporting the findings of this manuscript are available from the corresponding author upon request. The mass spectrometry raw files were deposited at MassIVE under accession number: MSV000082407 and at ProteomeXchange under accession number: PXD009923. The RNAseq data raw files were deposited at GEO and are accessible under accession number GSE115142.

## Electronic supplementary material

Supplementary Information
