# Supplementary material for: A widespread alternate form of cap-dependent mRNA translation initiation
Source: Nat Commun. 2018 Aug 3;9:3068. doi: 10.1038/s41467-018-05539-0 (PMC6076257; doi:10.1038/s41467-018-05539-0)
Supplement: Supplementary file 1 — Supplementary Information [file 41467_2018_5539_MOESM1_ESM.pdf]

Supplementary Figures for de la Parra et al.

Supplementary Figure 1

Supplementary Figure 2

Supplementary Figure 3

Supplementary Figure 4

Supplementary Figure 5

Supplementary Figure 6

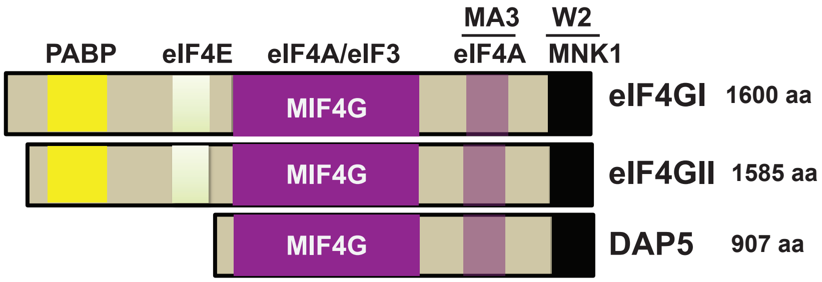

Supplementary Fig. 1 Domain structure of eIF4GI, eIF4GII and DAP5. The eIF4G family shares homology in the middle and C-terminal regions. The middle domain, the MIF4G domain, has binidng sites for eIF4AI and eIF3. The C-terminal domain contains a second eIF4AI binding domain, MA3, whereas the W2 domain motif and binds Mnk1, the eIF4E kinase. DAP5 lacks the N-terminal region found in eIF4GI and eIF4GII, and therefore cannot bind eIF4E or PABP.

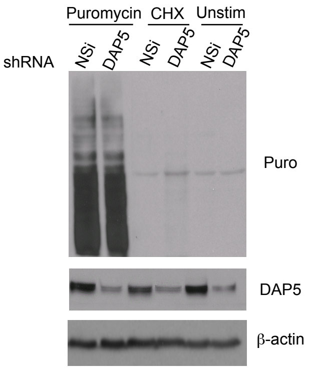

Supplementary Fig. 2 Puromycin labeling of nascent polypeptides in MDA-MB231 cells without and with DAP5-silencing. Dox-inducible TRIPZ RFP shRNA lentivirus vectors were used to silence DAP5 or express non-silencing (NSi) RNA. Representative immunoblot shown from at least three independent experiments with anti-puromycin, anti-DAP5 or anti-actin antibodies. Cyclohexamide (CHX) and unstimulated (Unstim) control cells served as a negative control.

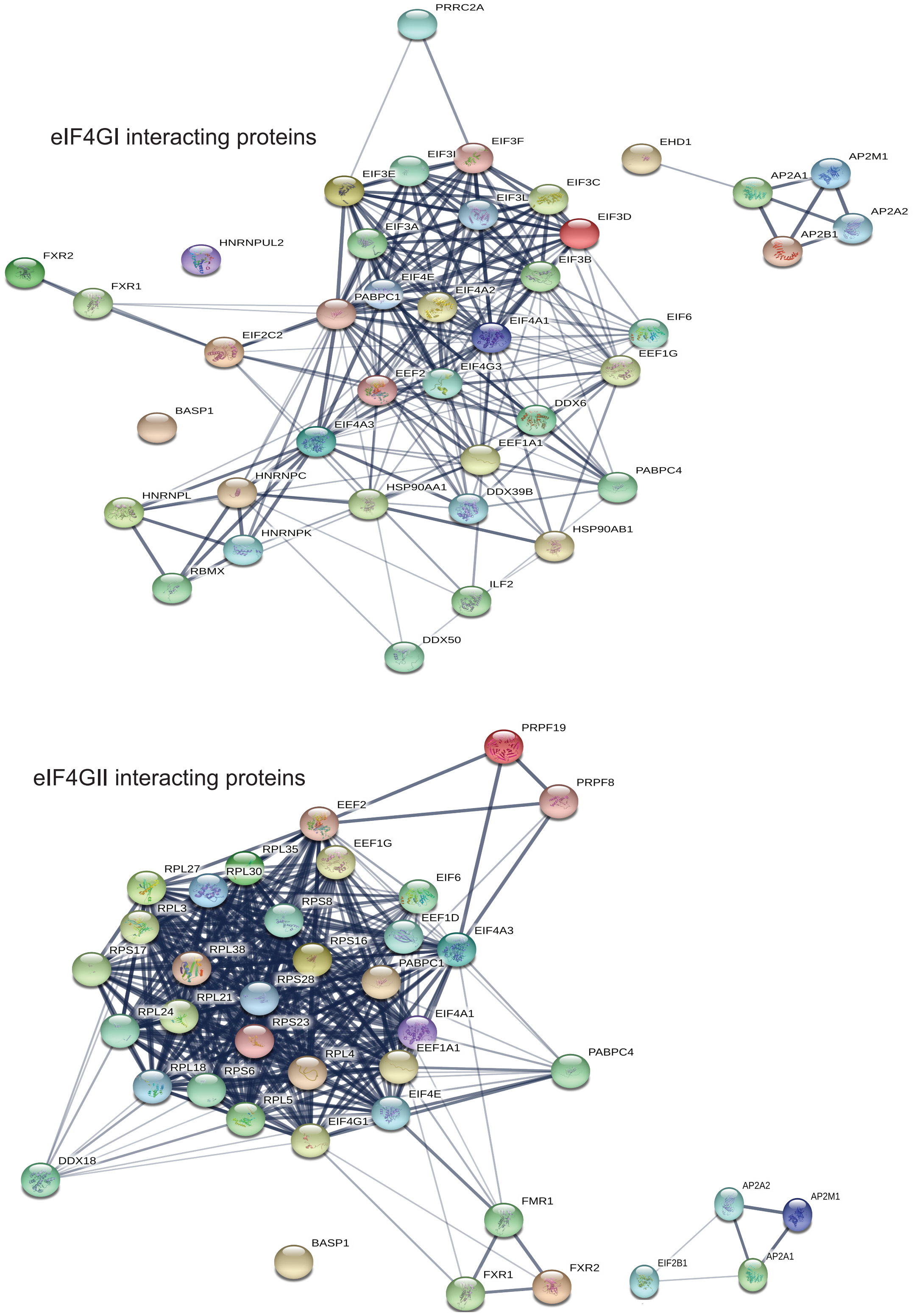

Supplementary Fig. 3 STRING analysis of protein interactions for eIF4GI and eIF4GII. Data drawn from Extended Data Table 2. Data analysis using SAINT algorithm of the top-ranked protein interactions.

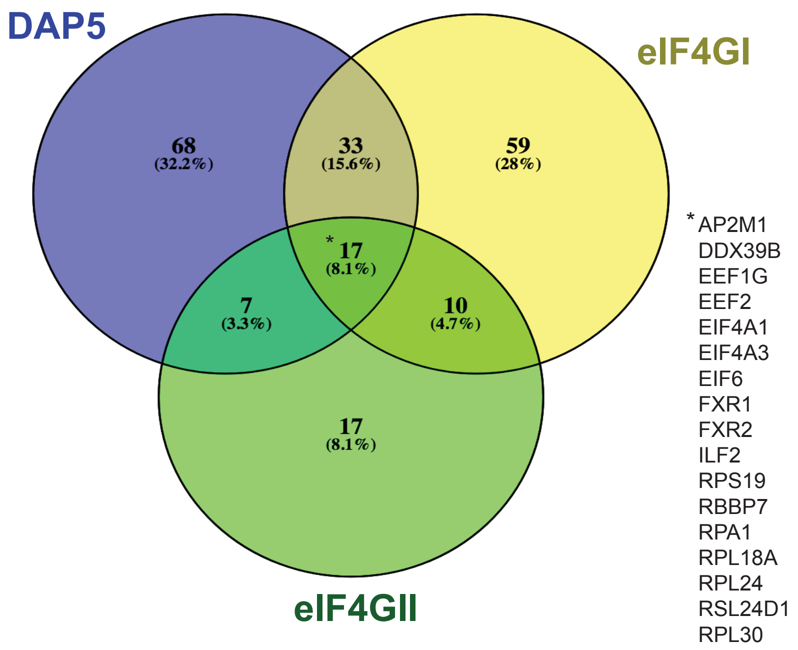

Supplementary Fig. 4 Comparison of proteins found in common or uniquely associated with eIF4GI, eIF4GII and/or DAP5. Results derived from mass spectrometry data showing proteins from the SAINT analysis. See also Supplementary Data 2 for soruce data used to develop the comparative data shown here. *, 17 proteins shared between eIF4GI, eIF4GII and DAP5.

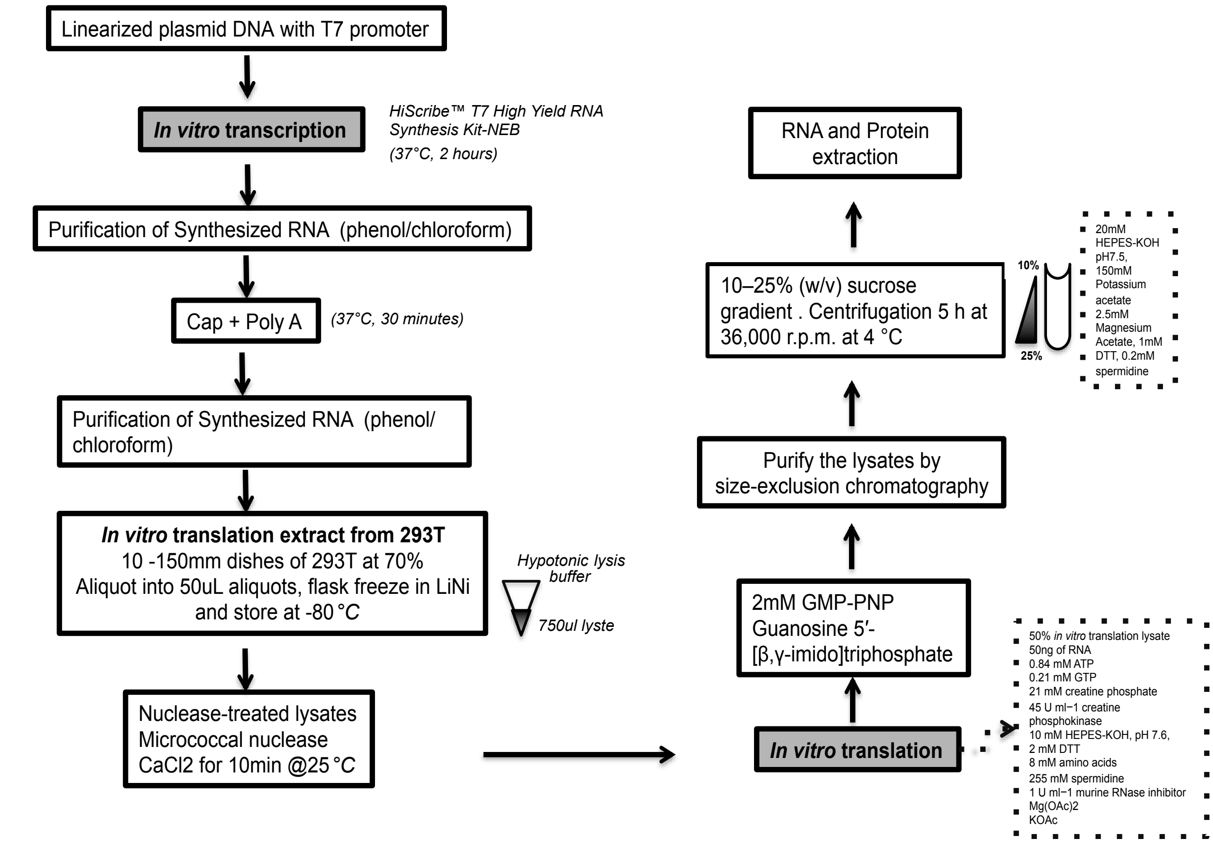

Supplementary Fig. 5 Flow chart outlining the preparation of in vitro 48S ribosome translation extracts. 293T cells were used for preparation of mRNA programmed cell free translation extracts with capped and polyadenylated mRNAs, followed by purification of 48S mRNA-40S ribosome initiation complexes.

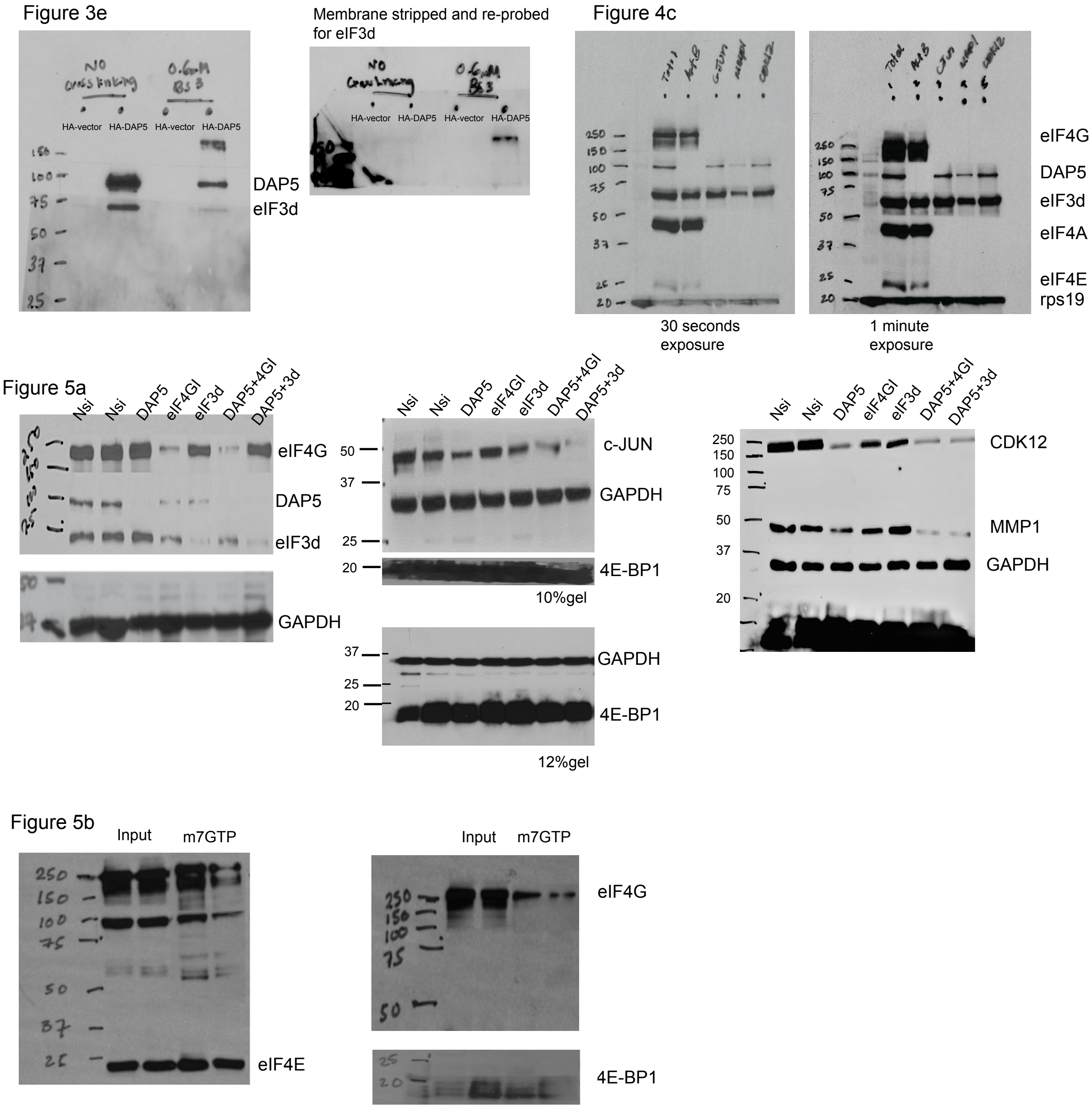

+

Supplementary Fig. 6. Uncropped scans of immunoblots shown in Figures 3e, 4c, 5a and 5b.
